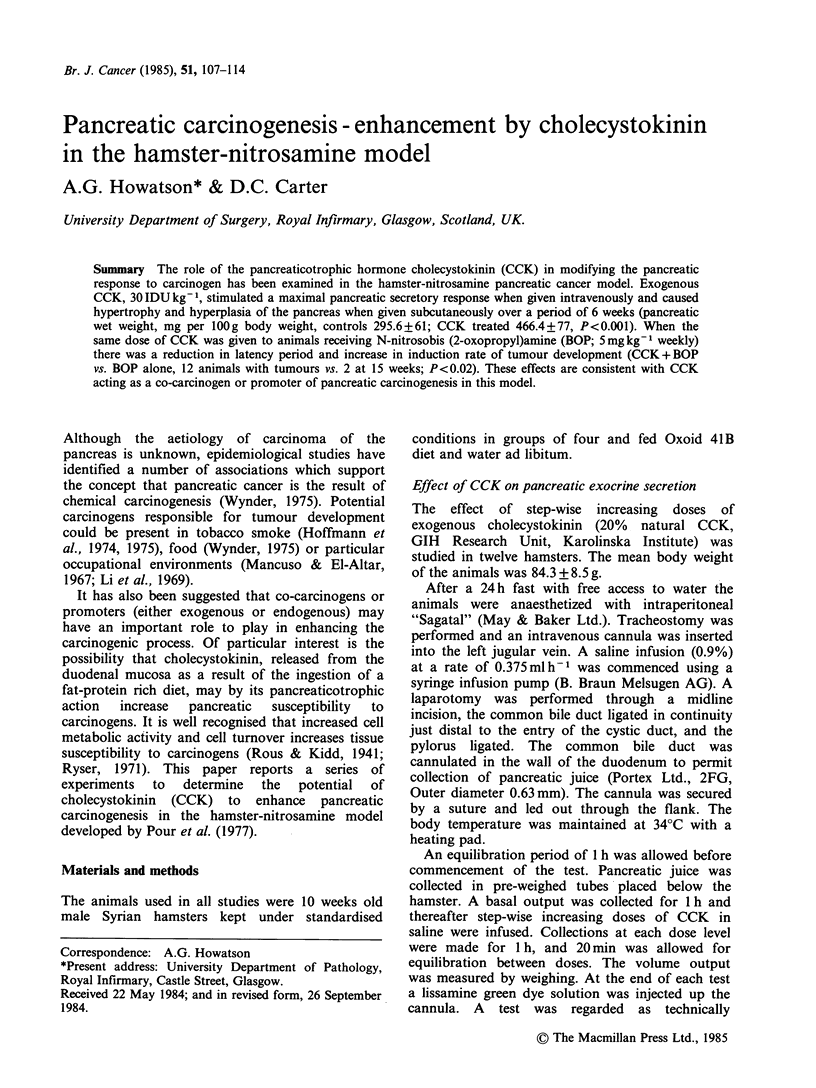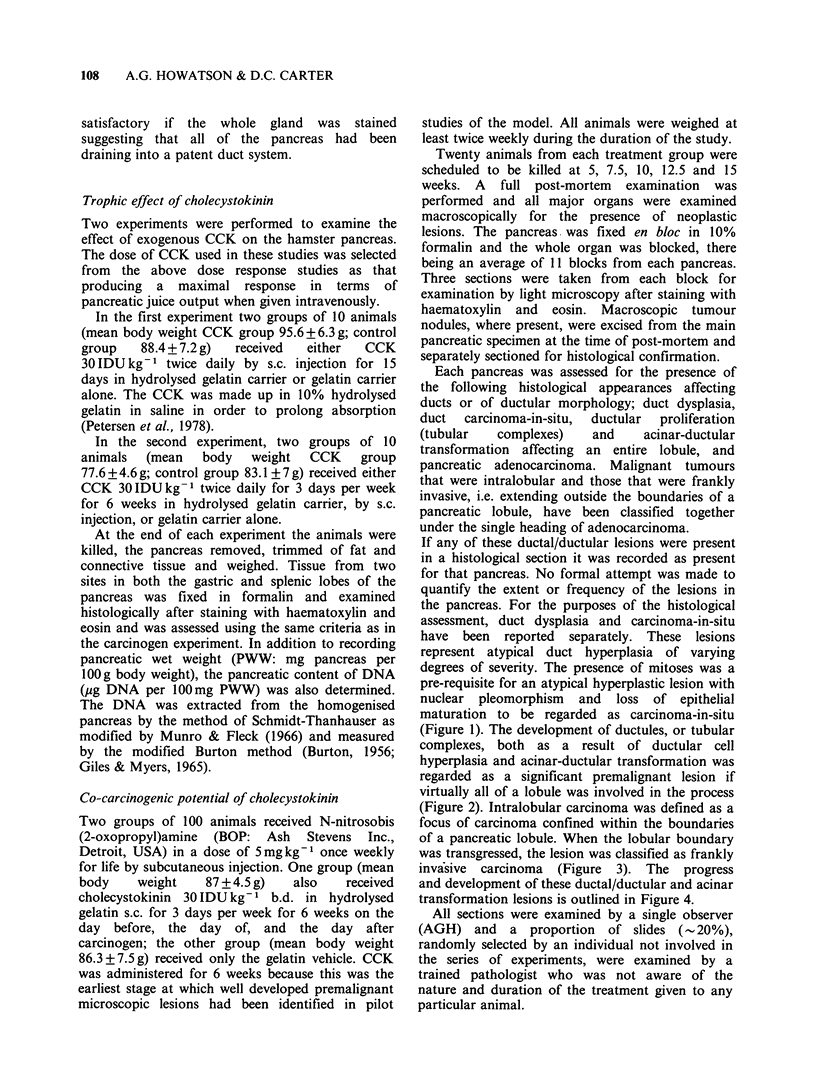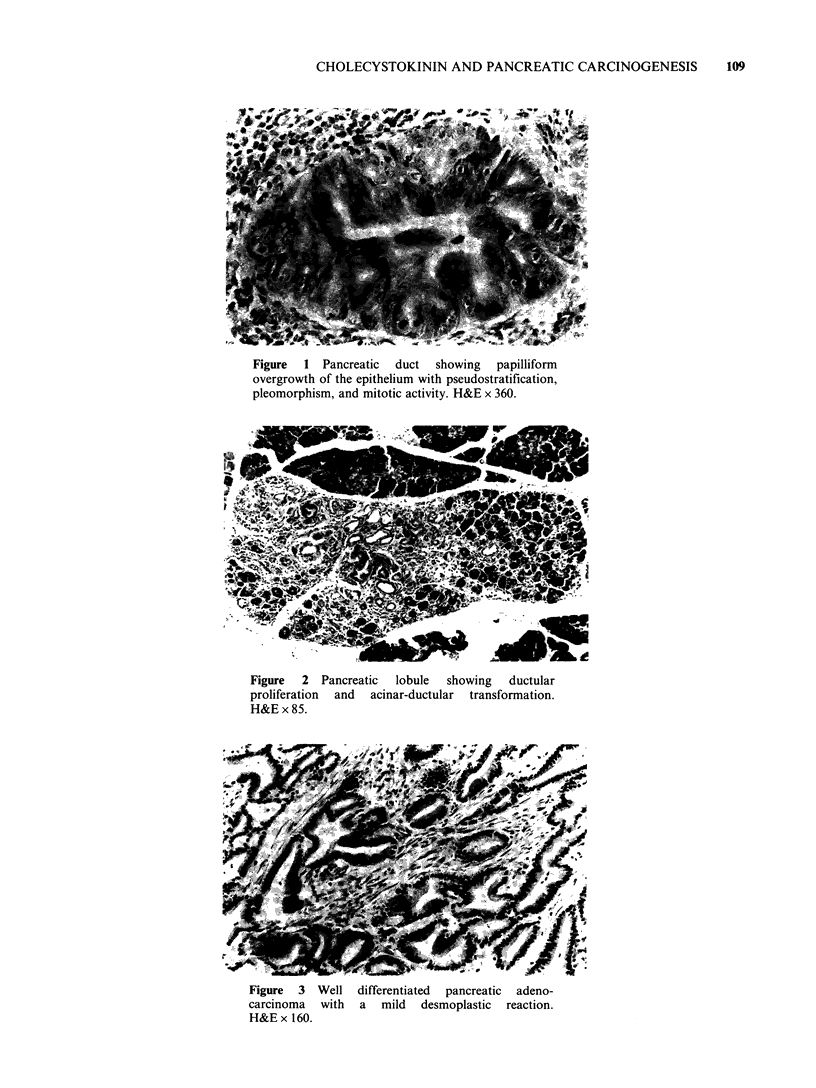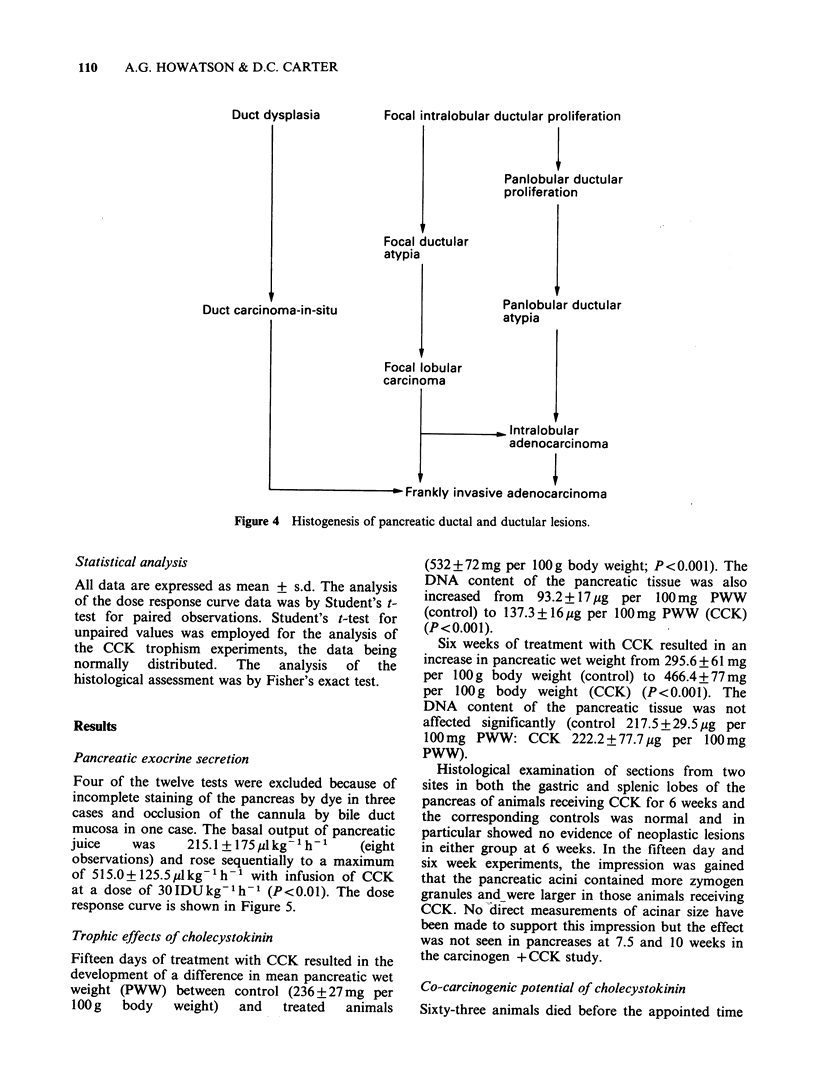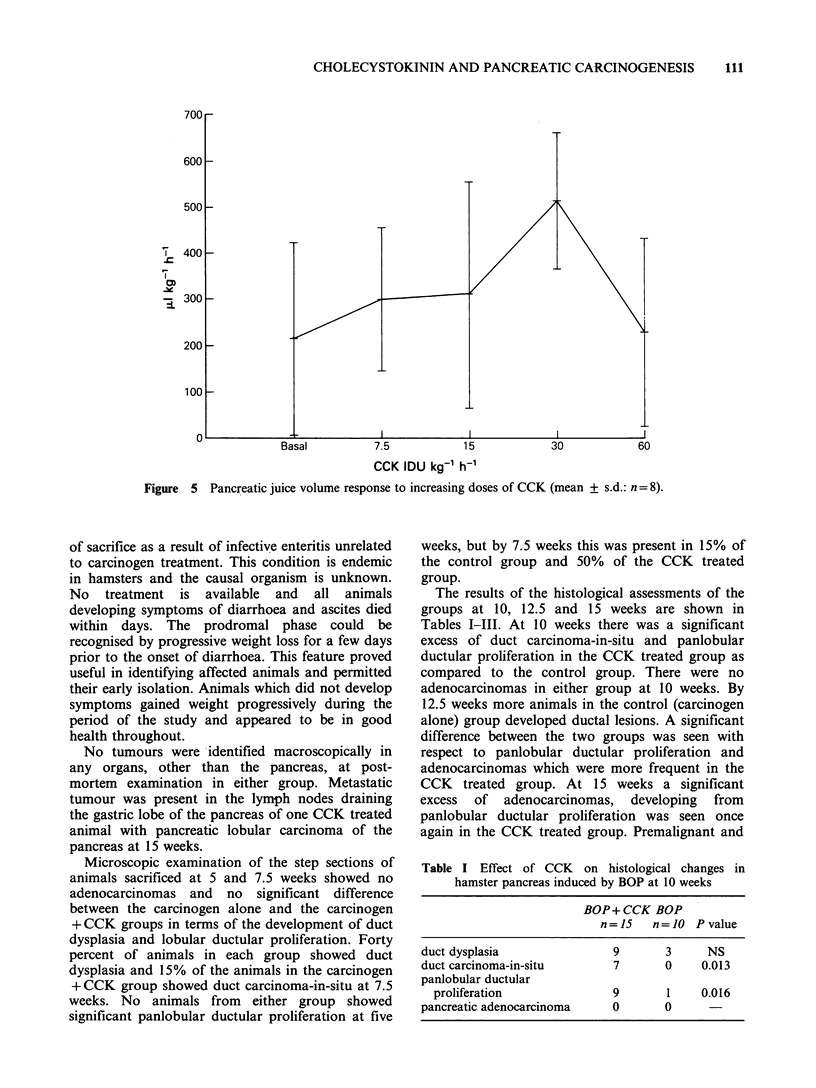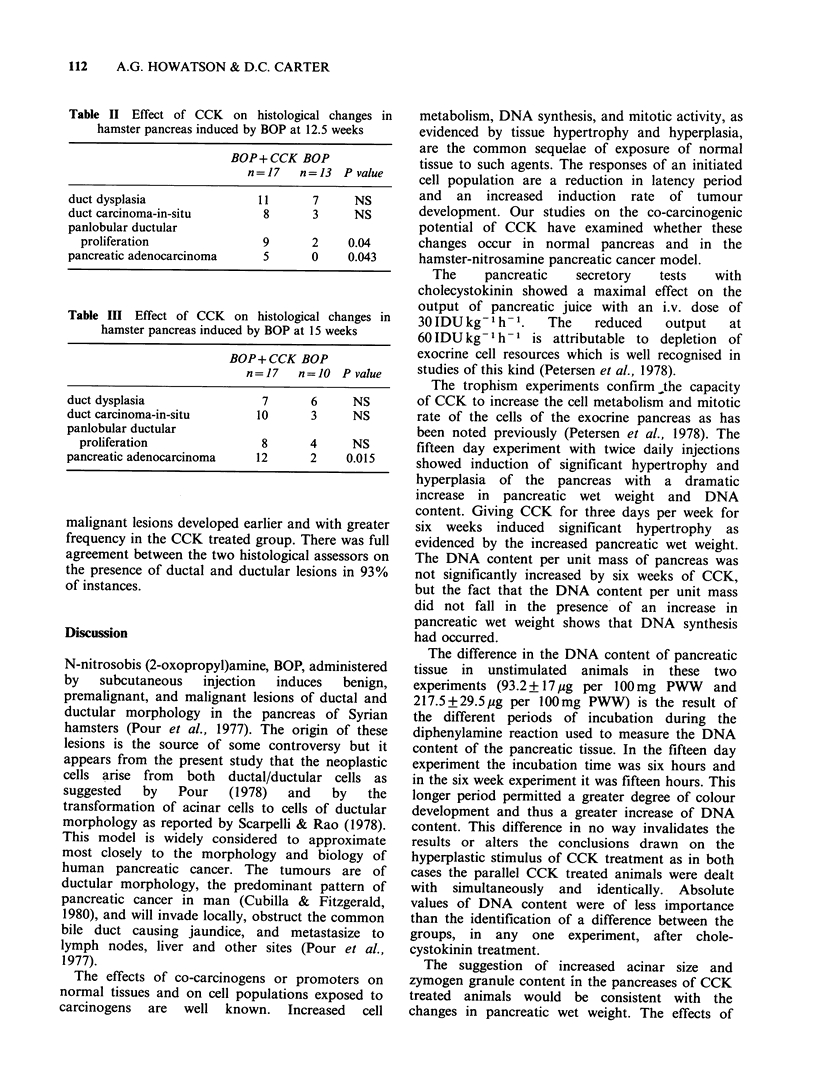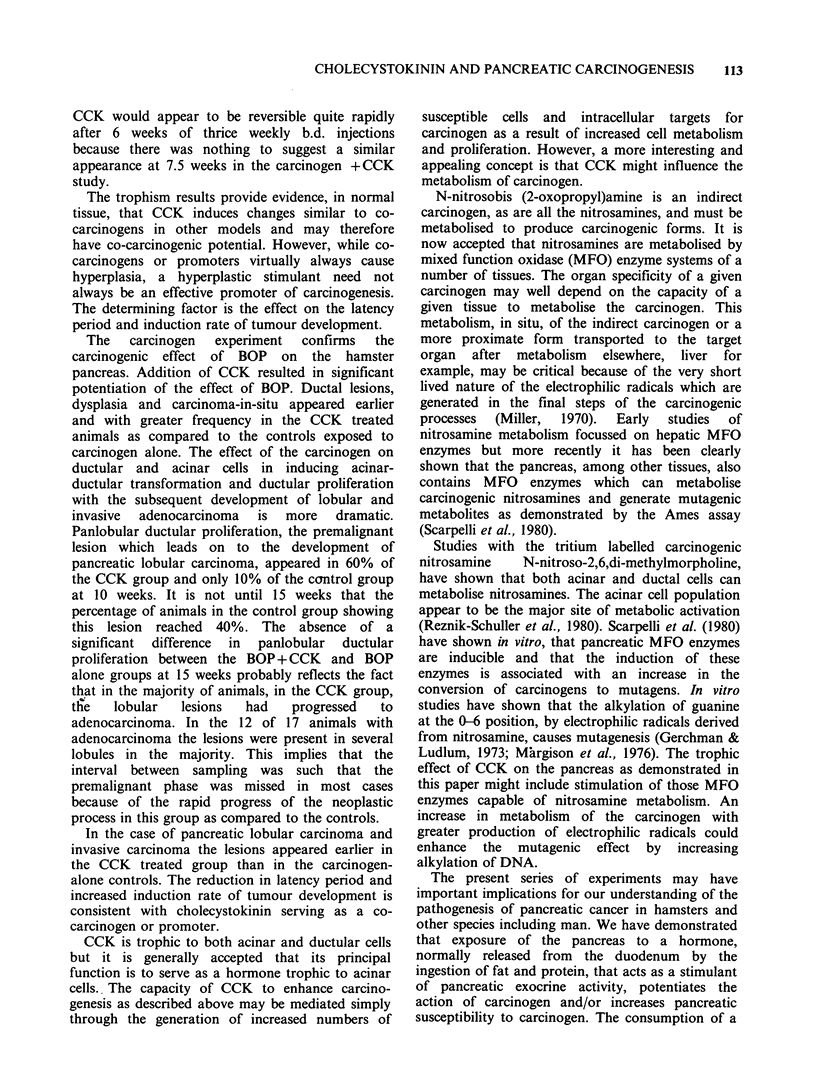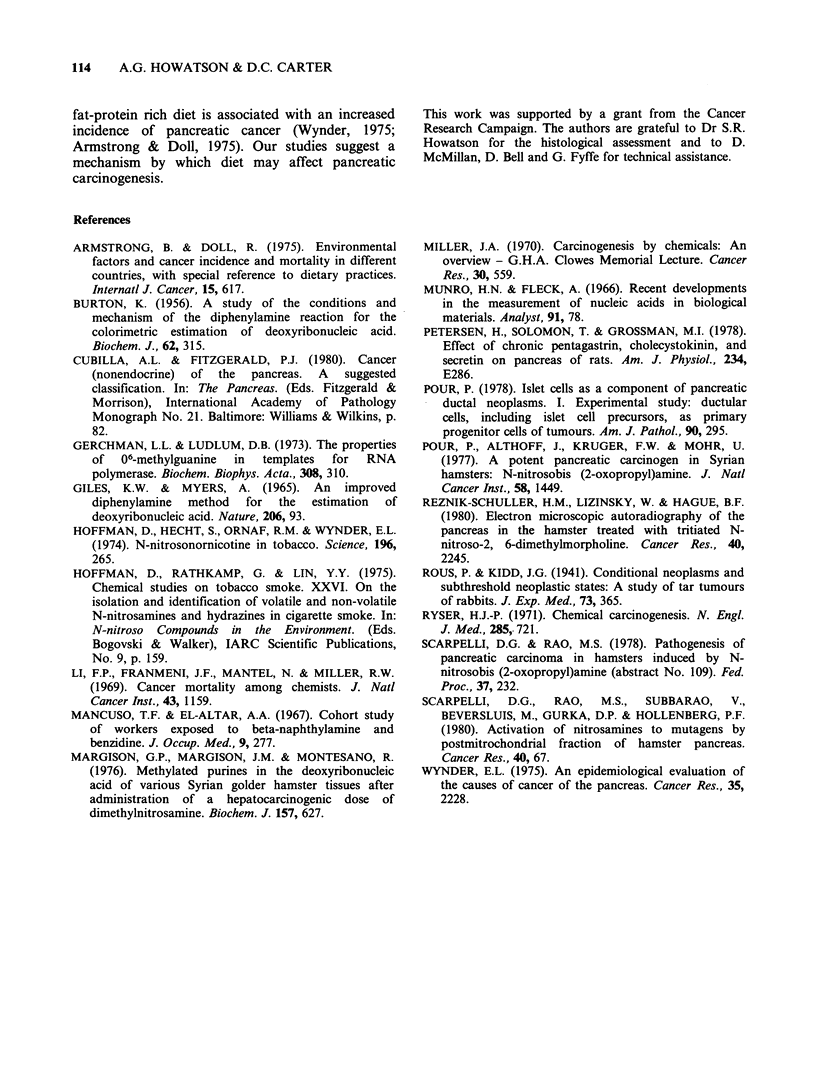# Pancreatic carcinogenesis-enhancement by cholecystokinin in the hamster-nitrosamine model.

**DOI:** 10.1038/bjc.1985.15

**Published:** 1985-01

**Authors:** A. G. Howatson, D. C. Carter

## Abstract

**Images:**


*Br. J. Cancer* (1985), **51**, 107–114

### Pancreatic carcinogenesis - enhancement by cholecystokinin in the hamster-nitrosamine model

A.G. Howatson* & D.C. Carter

*University Department of Surgery, Royal Infirmary, Glasgow, Scotland, UK.*

**Summary** The role of the pancreaticotrophic hormone cholecystokinin (CCK) in modifying the pancreatic response to carcinogen has been examined in the hamster-nitrosamine pancreatic cancer model. Exogenous CCK, 30 IDU $kg^{-1}$, stimulated a maximal pancreatic secretory response when given intravenously and caused hypertrophy and hyperplasia of the pancreas when given subcutaneously over a period of 6 weeks (pancreatic wet weight, mg per 100 g body weight, controls $295.6 \pm 61$; CCK treated $466.4 \pm 77$, $P < 0.001$). When the same dose of CCK was given to animals receiving N-nitrosobis (2-oxopropyl)amine (BOP; 5 mg $kg^{-1}$ weekly) there was a reduction in latency period and increase in induction rate of tumour development (CCK + BOP *vs.* BOP alone, 12 animals with tumours *vs.* 2 at 15 weeks; $P < 0.02$). These effects are consistent with CCK acting as a co-carcinogen or promoter of pancreatic carcinogenesis in this model.

Although the aetiology of carcinoma of the pancreas is unknown, epidemiological studies have identified a number of associations which support the concept that pancreatic cancer is the result of chemical carcinogenesis (Wynder, 1975). Potential carcinogens responsible for tumour development could be present in tobacco smoke (Hoffmann *et al.*, 1974, 1975), food (Wynder, 1975) or particular occupational environments (Mancuso & El-Altar, 1967; Li *et al.*, 1969).

It has also been suggested that co-carcinogens or promoters (either exogenous or endogenous) may have an important role to play in enhancing the carcinogenic process. Of particular interest is the possibility that cholecystokinin, released from the duodenal mucosa as a result of the ingestion of a fat-protein rich diet, may by its pancreaticotrophic action increase pancreatic susceptibility to carcinogens. It is well recognised that increased cell metabolic activity and cell turnover increases tissue susceptibility to carcinogens (Rous & Kidd, 1941; Ryser, 1971). This paper reports a series of experiments to determine the potential of cholecystokinin (CCK) to enhance pancreatic carcinogenesis in the hamster-nitrosamine model developed by Pour *et al.* (1977).

### Materials and methods

The animals used in all studies were 10 weeks old male Syrian hamsters kept under standardised conditions in groups of four and fed Oxoid 41B diet and water ad libitum.

### *Effect of CCK on pancreatic exocrine secretion*

The effect of step-wise increasing doses of exogenous cholecystokinin (20% natural CCK, GIH Research Unit, Karolinska Institute) was studied in twelve hamsters. The mean body weight of the animals was $84.3 \pm 8.5$ g.

After a 24 h fast with free access to water the animals were anaesthetized with intraperitoneal "Sagatal" (May & Baker Ltd.). Tracheostomy was performed and an intravenous cannula was inserted into the left jugular vein. A saline infusion (0.9%) at a rate of 0.375 ml $h^{-1}$ was commenced using a syringe infusion pump (B. Braun Melsugen AG). A laparotomy was performed through a midline incision, the common bile duct ligated in continuity just distal to the entry of the cystic duct, and the pylorus ligated. The common bile duct was cannulated in the wall of the duodenum to permit collection of pancreatic juice (Portex Ltd., 2FG, Outer diameter 0.63 mm). The cannula was secured by a suture and led out through the flank. The body temperature was maintained at 34°C with a heating pad.

An equilibration period of 1 h was allowed before commencement of the test. Pancreatic juice was collected in pre-weighed tubes placed below the hamster. A basal output was collected for 1 h and thereafter step-wise increasing doses of CCK in saline were infused. Collections at each dose level were made for 1 h, and 20 min was allowed for equilibration between doses. The volume output was measured by weighing. At the end of each test a lissamine green dye solution was injected up the cannula. A test was regarded as technically

Correspondence: A.G. Howatson
*Present address: University Department of Pathology, Royal Infirmary, Castle Street, Glasgow.
Received 22 May 1984; and in revised form, 26 September 1984.

© The Macmillan Press Ltd., 1985

satisfactory if the whole gland was stained suggesting that all of the pancreas had been draining into a patent duct system.

*Trophic effect of cholecystokinin*

Two experiments were performed to examine the effect of exogenous CCK on the hamster pancreas. The dose of CCK used in these studies was selected from the above dose response studies as that producing a maximal response in terms of pancreatic juice output when given intravenously.

In the first experiment two groups of 10 animals (mean body weight CCK group $95.6 \pm 6.3$ g; control group $88.4 \pm 7.2$ g) received either CCK 30 IDU $kg^{-1}$ twice daily by s.c. injection for 15 days in hydrolysed gelatin carrier or gelatin carrier alone. The CCK was made up in 10% hydrolysed gelatin in saline in order to prolong absorption (Petersen *et al.*, 1978).

In the second experiment, two groups of 10 animals (mean body weight CCK group $77.6 \pm 4.6$ g; control group $83.1 \pm 7$ g) received either CCK 30 IDU $kg^{-1}$ twice daily for 3 days per week for 6 weeks in hydrolysed gelatin carrier, by s.c. injection, or gelatin carrier alone.

At the end of each experiment the animals were killed, the pancreas removed, trimmed of fat and connective tissue and weighed. Tissue from two sites in both the gastric and splenic lobes of the pancreas was fixed in formalin and examined histologically after staining with haematoxylin and eosin and was assessed using the same criteria as in the carcinogen experiment. In addition to recording pancreatic wet weight (PWW: mg pancreas per 100 g body weight), the pancreatic content of DNA ($\mu$g DNA per 100 mg PWW) was also determined. The DNA was extracted from the homogenised pancreas by the method of Schmidt-Thanhauser as modified by Munro & Fleck (1966) and measured by the modified Burton method (Burton, 1956; Giles & Myers, 1965).

*Co-carcinogenic potential of cholecystokinin*

Two groups of 100 animals received N-nitrosobis (2-oxopropyl)amine (BOP: Ash Stevens Inc., Detroit, USA) in a dose of 5 mg $kg^{-1}$ once weekly for life by subcutaneous injection. One group (mean body weight $87 \pm 4.5$ g) also received cholecystokinin 30 IDU $kg^{-1}$ b.d. in hydrolysed gelatin s.c. for 3 days per week for 6 weeks on the day before, the day of, and the day after carcinogen; the other group (mean body weight $86.3 \pm 7.5$ g) received only the gelatin vehicle. CCK was administered for 6 weeks because this was the earliest stage at which well developed premalignant microscopic lesions had been identified in pilot studies of the model. All animals were weighed at least twice weekly during the duration of the study.

Twenty animals from each treatment group were scheduled to be killed at 5, 7.5, 10, 12.5 and 15 weeks. A full post-mortem examination was performed and all major organs were examined macroscopically for the presence of neoplastic lesions. The pancreas was fixed *en bloc* in 10% formalin and the whole organ was blocked, there being an average of 11 blocks from each pancreas. Three sections were taken from each block for examination by light microscopy after staining with haematoxylin and eosin. Macroscopic tumour nodules, where present, were excised from the main pancreatic specimen at the time of post-mortem and separately sectioned for histological confirmation.

Each pancreas was assessed for the presence of the following histological appearances affecting ducts or of ductular morphology; duct dysplasia, duct carcinoma-in-situ, ductular proliferation (tubular complexes) and acinar-ductular transformation affecting an entire lobule, and pancreatic adenocarcinoma. Malignant tumours that were intralobular and those that were frankly invasive, i.e. extending outside the boundaries of a pancreatic lobule, have been classified together under the single heading of adenocarcinoma.

If any of these ductal/ductular lesions were present in a histological section it was recorded as present for that pancreas. No formal attempt was made to quantify the extent or frequency of the lesions in the pancreas. For the purposes of the histological assessment, duct dysplasia and carcinoma-in-situ have been reported separately. These lesions represent atypical duct hyperplasia of varying degrees of severity. The presence of mitoses was a pre-requisite for an atypical hyperplastic lesion with nuclear pleomorphism and loss of epithelial maturation to be regarded as carcinoma-in-situ (Figure 1). The development of ductules, or tubular complexes, both as a result of ductular cell hyperplasia and acinar-ductular transformation was regarded as a significant premalignant lesion if virtually all of a lobule was involved in the process (Figure 2). Intralobular carcinoma was defined as a focus of carcinoma confined within the boundaries of a pancreatic lobule. When the lobular boundary was transgressed, the lesion was classified as frankly invasive carcinoma (Figure 3). The progress and development of these ductal/ductular and acinar transformation lesions is outlined in Figure 4.

All sections were examined by a single observer (AGH) and a proportion of slides ($\sim$20%), randomly selected by an individual not involved in the series of experiments, were examined by a trained pathologist who was not aware of the nature and duration of the treatment given to any particular animal.

**Figure 1** Pancreatic duct showing papilliform overgrowth of the epithelium with pseudostratification, pleomorphism, and mitotic activity. H&E × 360.

**Figure 2** Pancreatic lobule showing ductular proliferation and acinar-ductular transformation. H&E × 85.

**Figure 3** Well differentiated pancreatic adenocarcinoma with a mild desmoplastic reaction. H&E × 160.

**Figure 4** Histogenesis of pancreatic ductal and ductular lesions.

*Statistical analysis*

All data are expressed as mean ± s.d. The analysis of the dose response curve data was by Student's *t*-test for paired observations. Student's *t*-test for unpaired values was employed for the analysis of the CCK trophism experiments, the data being normally distributed. The analysis of the histological assessment was by Fisher's exact test.

### Results

*Pancreatic exocrine secretion*

Four of the twelve tests were excluded because of incomplete staining of the pancreas by dye in three cases and occlusion of the cannula by bile duct mucosa in one case. The basal output of pancreatic juice was $215.1 \pm 175\,\mu l\,kg^{-1}\,h^{-1}$ (eight observations) and rose sequentially to a maximum of $515.0 \pm 125.5\,\mu l\,kg^{-1}\,h^{-1}$ with infusion of CCK at a dose of $30\,IDU\,kg^{-1}\,h^{-1}$ ($P<0.01$). The dose response curve is shown in Figure 5.

*Trophic effects of cholecystokinin*

Fifteen days of treatment with CCK resulted in the development of a difference in mean pancreatic wet weight (PWW) between control ($236 \pm 27$ mg per 100 g body weight) and treated animals ($532 \pm 72$ mg per 100 g body weight; $P<0.001$). The DNA content of the pancreatic tissue was also increased from $93.2 \pm 17\,\mu g$ per 100 mg PWW (control) to $137.3 \pm 16\,\mu g$ per 100 mg PWW (CCK) ($P<0.001$).

Six weeks of treatment with CCK resulted in an increase in pancreatic wet weight from $295.6 \pm 61$ mg per 100 g body weight (control) to $466.4 \pm 77$ mg per 100 g body weight (CCK) ($P<0.001$). The DNA content of the pancreatic tissue was not affected significantly (control $217.5 \pm 29.5\,\mu g$ per 100 mg PWW: CCK $222.2 \pm 77.7\,\mu g$ per 100 mg PWW).

Histological examination of sections from two sites in both the gastric and splenic lobes of the pancreas of animals receiving CCK for 6 weeks and the corresponding controls was normal and in particular showed no evidence of neoplastic lesions in either group at 6 weeks. In the fifteen day and six week experiments, the impression was gained that the pancreatic acini contained more zymogen granules and were larger in those animals receiving CCK. No direct measurements of acinar size have been made to support this impression but the effect was not seen in pancreases at 7.5 and 10 weeks in the carcinogen + CCK study.

*Co-carcinogenic potential of cholecystokinin*

Sixty-three animals died before the appointed time

**Figure 5** Pancreatic juice volume response to increasing doses of CCK (mean $\pm$ s.d.: $n=8$).

of sacrifice as a result of infective enteritis unrelated to carcinogen treatment. This condition is endemic in hamsters and the causal organism is unknown. No treatment is available and all animals developing symptoms of diarrhoea and ascites died within days. The prodromal phase could be recognised by progressive weight loss for a few days prior to the onset of diarrhoea. This feature proved useful in identifying affected animals and permitted their early isolation. Animals which did not develop symptoms gained weight progressively during the period of the study and appeared to be in good health throughout.

No tumours were identified macroscopically in any organs, other than the pancreas, at post-mortem examination in either group. Metastatic tumour was present in the lymph nodes draining the gastric lobe of the pancreas of one CCK treated animal with pancreatic lobular carcinoma of the pancreas at 15 weeks.

Microscopic examination of the step sections of animals sacrificed at 5 and 7.5 weeks showed no adenocarcinomas and no significant difference between the carcinogen alone and the carcinogen +CCK groups in terms of the development of duct dysplasia and lobular ductular proliferation. Forty percent of animals in each group showed duct dysplasia and 15% of the animals in the carcinogen +CCK group showed duct carcinoma-in-situ at 7.5 weeks. No animals from either group showed significant panlobular ductular proliferation at five weeks, but by 7.5 weeks this was present in 15% of the control group and 50% of the CCK treated group.

The results of the histological assessments of the groups at 10, 12.5 and 15 weeks are shown in Tables I–III. At 10 weeks there was a significant excess of duct carcinoma-in-situ and panlobular ductular proliferation in the CCK treated group as compared to the control group. There were no adenocarcinomas in either group at 10 weeks. By 12.5 weeks more animals in the control (carcinogen alone) group developed ductal lesions. A significant difference between the two groups was seen with respect to panlobular ductular proliferation and adenocarcinomas which were more frequent in the CCK treated group. At 15 weeks a significant excess of adenocarcinomas, developing from panlobular ductular proliferation was seen once again in the CCK treated group. Premalignant and

**Table I** Effect of CCK on histological changes in hamster pancreas induced by BOP at 10 weeks

| | *BOP+CCK* *n=15* | *BOP* *n=10* | *P* value |
|---|---|---|---|
| duct dysplasia | 9 | 3 | NS |
| duct carcinoma-in-situ | 7 | 0 | 0.013 |
| panlobular ductular proliferation | 9 | 1 | 0.016 |
| pancreatic adenocarcinoma | 0 | 0 | — |

**Table II** Effect of CCK on histological changes in hamster pancreas induced by BOP at 12.5 weeks

| | *BOP+CCK* *n=17* | *BOP* *n=13* | *P value* |
|---|---|---|---|
| duct dysplasia | 11 | 7 | NS |
| duct carcinoma-in-situ | 8 | 3 | NS |
| panlobular ductular proliferation | 9 | 2 | 0.04 |
| pancreatic adenocarcinoma | 5 | 0 | 0.043 |

**Table III** Effect of CCK on histological changes in hamster pancreas induced by BOP at 15 weeks

| | *BOP+CCK* *n=17* | *BOP* *n=10* | *P value* |
|---|---|---|---|
| duct dysplasia | 7 | 6 | NS |
| duct carcinoma-in-situ | 10 | 3 | NS |
| panlobular ductular proliferation | 8 | 4 | NS |
| pancreatic adenocarcinoma | 12 | 2 | 0.015 |

malignant lesions developed earlier and with greater frequency in the CCK treated group. There was full agreement between the two histological assessors on the presence of ductal and ductular lesions in 93% of instances.

### Discussion

N-nitrosobis (2-oxopropyl)amine, BOP, administered by subcutaneous injection induces benign, premalignant, and malignant lesions of ductal and ductular morphology in the pancreas of Syrian hamsters (Pour *et al.*, 1977). The origin of these lesions is the source of some controversy but it appears from the present study that the neoplastic cells arise from both ductal/ductular cells as suggested by Pour (1978) and by the transformation of acinar cells to cells of ductular morphology as reported by Scarpelli & Rao (1978). This model is widely considered to approximate most closely to the morphology and biology of human pancreatic cancer. The tumours are of ductular morphology, the predominant pattern of pancreatic cancer in man (Cubilla & Fitzgerald, 1980), and will invade locally, obstruct the common bile duct causing jaundice, and metastasize to lymph nodes, liver and other sites (Pour *et al.*, 1977).

The effects of co-carcinogens or promoters on normal tissues and on cell populations exposed to carcinogens are well known. Increased cell metabolism, DNA synthesis, and mitotic activity, as evidenced by tissue hypertrophy and hyperplasia, are the common sequelae of exposure of normal tissue to such agents. The responses of an initiated cell population are a reduction in latency period and an increased induction rate of tumour development. Our studies on the co-carcinogenic potential of CCK have examined whether these changes occur in normal pancreas and in the hamster-nitrosamine pancreatic cancer model.

The pancreatic secretory tests with cholecystokinin showed a maximal effect on the output of pancreatic juice with an i.v. dose of 30 IDU $kg^{-1}h^{-1}$. The reduced output at 60 IDU $kg^{-1}h^{-1}$ is attributable to depletion of exocrine cell resources which is well recognised in studies of this kind (Petersen *et al.*, 1978).

The trophism experiments confirm the capacity of CCK to increase the cell metabolism and mitotic rate of the cells of the exocrine pancreas as has been noted previously (Petersen *et al.*, 1978). The fifteen day experiment with twice daily injections showed induction of significant hypertrophy and hyperplasia of the pancreas with a dramatic increase in pancreatic wet weight and DNA content. Giving CCK for three days per week for six weeks induced significant hypertrophy as evidenced by the increased pancreatic wet weight. The DNA content per unit mass of pancreas was not significantly increased by six weeks of CCK, but the fact that the DNA content per unit mass did not fall in the presence of an increase in pancreatic wet weight shows that DNA synthesis had occurred.

The difference in the DNA content of pancreatic tissue in unstimulated animals in these two experiments ($93.2 \pm 17\,\mu g$ per 100 mg PWW and $217.5 \pm 29.5\,\mu g$ per 100 mg PWW) is the result of the different periods of incubation during the diphenylamine reaction used to measure the DNA content of the pancreatic tissue. In the fifteen day experiment the incubation time was six hours and in the six week experiment it was fifteen hours. This longer period permitted a greater degree of colour development and thus a greater increase of DNA content. This difference in no way invalidates the results or alters the conclusions drawn on the hyperplastic stimulus of CCK treatment as in both cases the parallel CCK treated animals were dealt with simultaneously and identically. Absolute values of DNA content were of less importance than the identification of a difference between the groups, in any one experiment, after cholecystokinin treatment.

The suggestion of increased acinar size and zymogen granule content in the pancreases of CCK treated animals would be consistent with the changes in pancreatic wet weight. The effects of

CCK would appear to be reversible quite rapidly after 6 weeks of thrice weekly b.d. injections because there was nothing to suggest a similar appearance at 7.5 weeks in the carcinogen +CCK study.

The trophism results provide evidence, in normal tissue, that CCK induces changes similar to co-carcinogens in other models and may therefore have co-carcinogenic potential. However, while co-carcinogens or promoters virtually always cause hyperplasia, a hyperplastic stimulant need not always be an effective promoter of carcinogenesis. The determining factor is the effect on the latency period and induction rate of tumour development.

The carcinogen experiment confirms the carcinogenic effect of BOP on the hamster pancreas. Addition of CCK resulted in significant potentiation of the effect of BOP. Ductal lesions, dysplasia and carcinoma-in-situ appeared earlier and with greater frequency in the CCK treated animals as compared to the controls exposed to carcinogen alone. The effect of the carcinogen on ductular and acinar cells in inducing acinar-ductular transformation and ductular proliferation with the subsequent development of lobular and invasive adenocarcinoma is more dramatic. Panlobular ductular proliferation, the premalignant lesion which leads on to the development of pancreatic lobular carcinoma, appeared in 60% of the CCK group and only 10% of the control group at 10 weeks. It is not until 15 weeks that the percentage of animals in the control group showing this lesion reached 40%. The absence of a significant difference in panlobular ductular proliferation between the BOP+CCK and BOP alone groups at 15 weeks probably reflects the fact that in the majority of animals, in the CCK group, the lobular lesions had progressed to adenocarcinoma. In the 12 of 17 animals with adenocarcinoma the lesions were present in several lobules in the majority. This implies that the interval between sampling was such that the premalignant phase was missed in most cases because of the rapid progress of the neoplastic process in this group as compared to the controls.

In the case of pancreatic lobular carcinoma and invasive carcinoma the lesions appeared earlier in the CCK treated group than in the carcinogen-alone controls. The reduction in latency period and increased induction rate of tumour development is consistent with cholecystokinin serving as a co-carcinogen or promoter.

CCK is trophic to both acinar and ductular cells but it is generally accepted that its principal function is to serve as a hormone trophic to acinar cells. The capacity of CCK to enhance carcinogenesis as described above may be mediated simply through the generation of increased numbers of susceptible cells and intracellular targets for carcinogen as a result of increased cell metabolism and proliferation. However, a more interesting and appealing concept is that CCK might influence the metabolism of carcinogen.

N-nitrosobis (2-oxopropyl)amine is an indirect carcinogen, as are all the nitrosamines, and must be metabolised to produce carcinogenic forms. It is now accepted that nitrosamines are metabolised by mixed function oxidase (MFO) enzyme systems of a number of tissues. The organ specificity of a given carcinogen may well depend on the capacity of a given tissue to metabolise the carcinogen. This metabolism, in situ, of the indirect carcinogen or a more proximate form transported to the target organ after metabolism elsewhere, liver for example, may be critical because of the very short lived nature of the electrophilic radicals which are generated in the final steps of the carcinogenic processes (Miller, 1970). Early studies of nitrosamine metabolism focussed on hepatic MFO enzymes but more recently it has been clearly shown that the pancreas, among other tissues, also contains MFO enzymes which can metabolise carcinogenic nitrosamines and generate mutagenic metabolites as demonstrated by the Ames assay (Scarpelli *et al.*, 1980).

Studies with the tritium labelled carcinogenic nitrosamine N-nitroso-2,6,di-methylmorpholine, have shown that both acinar and ductal cells can metabolise nitrosamines. The acinar cell population appear to be the major site of metabolic activation (Reznik-Schuller *et al.*, 1980). Scarpelli *et al.* (1980) have shown *in vitro*, that pancreatic MFO enzymes are inducible and that the induction of these enzymes is associated with an increase in the conversion of carcinogens to mutagens. *In vitro* studies have shown that the alkylation of guanine at the 0–6 position, by electrophilic radicals derived from nitrosamine, causes mutagenesis (Gerchman & Ludlum, 1973; Margison *et al.*, 1976). The trophic effect of CCK on the pancreas as demonstrated in this paper might include stimulation of those MFO enzymes capable of nitrosamine metabolism. An increase in metabolism of the carcinogen with greater production of electrophilic radicals could enhance the mutagenic effect by increasing alkylation of DNA.

The present series of experiments may have important implications for our understanding of the pathogenesis of pancreatic cancer in hamsters and other species including man. We have demonstrated that exposure of the pancreas to a hormone, normally released from the duodenum by the ingestion of fat and protein, that acts as a stimulant of pancreatic exocrine activity, potentiates the action of carcinogen and/or increases pancreatic susceptibility to carcinogen. The consumption of a fat-protein rich diet is associated with an increased incidence of pancreatic cancer (Wynder, 1975; Armstrong & Doll, 1975). Our studies suggest a mechanism by which diet may affect pancreatic carcinogenesis.

This work was supported by a grant from the Cancer Research Campaign. The authors are grateful to Dr S.R. Howatson for the histological assessment and to D. McMillan, D. Bell and G. Fyffe for technical assistance.

**References**

ARMSTRONG, B. & DOLL, R. (1975). Environmental factors and cancer incidence and mortality in different countries, with special reference to dietary practices. *Internatl J. Cancer*, **15**, 617.

BURTON, K. (1956). A study of the conditions and mechanism of the diphenylamine reaction for the colorimetric estimation of deoxyribonucleic acid. *Biochem. J.*, **62**, 315.

CUBILLA, A.L. & FITZGERALD, P.J. (1980). Cancer (nonendocrine) of the pancreas. A suggested classification. In: *The Pancreas*. (Eds. Fitzgerald & Morrison), International Academy of Pathology Monograph No. 21. Baltimore: Williams & Wilkins, p. 82.

GERCHMAN, L.L. & LUDLUM, D.B. (1973). The properties of $0^6$-methylguanine in templates for RNA polymerase. *Biochem. Biophys. Acta.*, **308**, 310.

GILES, K.W. & MYERS, A. (1965). An improved diphenylamine method for the estimation of deoxyribonucleic acid. *Nature*, **206**, 93.

HOFFMAN, D., HECHT, S., ORNAF, R.M. & WYNDER, E.L. (1974). N-nitrosonornicotine in tobacco. *Science*, **196**, 265.

HOFFMAN, D., RATHKAMP, G. & LIN, Y.Y. (1975). Chemical studies on tobacco smoke. XXVI. On the isolation and identification of volatile and non-volatile N-nitrosamines and hydrazines in cigarette smoke. In: *N-nitroso Compounds in the Environment*. (Eds. Bogovski & Walker), IARC Scientific Publications, No. 9, p. 159.

LI, F.P., FRANMENI, J.F., MANTEL, N. & MILLER, R.W. (1969). Cancer mortality among chemists. *J. Natl Cancer Inst.*, **43**, 1159.

MANCUSO, T.F. & EL-ALTAR, A.A. (1967). Cohort study of workers exposed to beta-naphthylamine and benzidine. *J. Occup. Med.*, **9**, 277.

MARGISON, G.P., MARGISON, J.M. & MONTESANO, R. (1976). Methylated purines in the deoxyribonucleic acid of various Syrian golder hamster tissues after administration of a hepatocarcinogenic dose of dimethylnitrosamine. *Biochem. J.* **157**, 627.

MILLER, J.A. (1970). Carcinogenesis by chemicals: An overview – G.H.A. Clowes Memorial Lecture. *Cancer Res.*, **30**, 559.

MUNRO, H.N. & FLECK, A. (1966). Recent developments in the measurement of nucleic acids in biological materials. *Analyst*, **91**, 78.

PETERSEN, H., SOLOMON, T. & GROSSMAN, M.I. (1978). Effect of chronic pentagastrin, cholecystokinin, and secretin on pancreas of rats. *Am. J. Physiol.*, **234**, E286.

POUR, P. (1978). Islet cells as a component of pancreatic ductal neoplasms. I. Experimental study: ductular cells, including islet cell precursors, as primary progenitor cells of tumours. *Am. J. Pathol.*, **90**, 295.

POUR, P., ALTHOFF, J., KRUGER, F.W. & MOHR, U. (1977). A potent pancreatic carcinogen in Syrian hamsters: N-nitrosobis (2-oxopropyl)amine. *J. Natl Cancer Inst.*, **58**, 1449.

REZNIK-SCHULLER, H.M., LIZINSKY, W. & HAGUE, B.F. (1980). Electron microscopic autoradiography of the pancreas in the hamster treated with tritiated N-nitroso-2, 6-dimethylmorpholine. *Cancer Res.*, **40**, 2245.

ROUS, P. & KIDD, J.G. (1941). Conditional neoplasms and subthreshold neoplastic states: A study of tar tumours of rabbits. *J. Exp. Med.*, **73**, 365.

RYSER, H.J.-P. (1971). Chemical carcinogenesis. *N. Engl. J. Med.*, **285**, 721.

SCARPELLI, D.G. & RAO, M.S. (1978). Pathogenesis of pancreatic carcinoma in hamsters induced by N-nitrosobis (2-oxopropyl)amine (abstract No. 109). *Fed. Proc.*, **37**, 232.

SCARPELLI, D.G., RAO, M.S., SUBBARAO, V., BEVERSLUIS, M., GURKA, D.P. & HOLLENBERG, P.F. (1980). Activation of nitrosamines to mutagens by postmitrochondrial fraction of hamster pancreas. *Cancer Res.*, **40**, 67.

WYNDER, E.L. (1975). An epidemiological evaluation of the causes of cancer of the pancreas. *Cancer Res.*, **35**, 2228.